# Recent Advancements in Serum Albumin-Based Nanovehicles Toward Potential Cancer Diagnosis and Therapy

**DOI:** 10.3389/fchem.2021.746646

**Published:** 2021-11-18

**Authors:** Xue Shen, Xiyang Liu, Tingting Li, Yin Chen, Yang Chen, Pan Wang, Lin Zheng, Hong Yang, Chunhui Wu, Shengqi Deng, Yiyao Liu

**Affiliations:** ^1^ Engineering Research Center for Pharmaceuticals and Equipments of Sichuan Province, Sichuan Industrial Institute of Antibiotics, School of Pharmacy, Chengdu University, Chengdu, China; ^2^ School of Life Science and Technology, University of Electronic Science and Technology of China, Chengdu, China; ^3^ State Key Laboratory of Trauma, Burns and Combined Injury, Institute of Combined Injury of PLA, Chongqing Engineering Research Center for Nanomedicine, College of Preventive Medicine, Third Military Medical University, Chongqing, China; ^4^ School of Mechanical Engineering, Chengdu University, Chengdu, China; ^5^ TCM Regulating Metabolic Diseases Key Laboratory of Sichuan Province, Hospital of Chengdu University of Traditional Chinese Medicine, Chengdu, China

**Keywords:** serum albumin-based nanovehicles, drug loading, diagnostic nanoprobes, cancer therapy, theranostics

## Abstract

Recently, drug delivery vehicles based on nanotechnology have significantly attracted the attention of researchers in the field of nanomedicine since they can achieve ideal drug release and biodistribution. Among the various organic or inorganic materials that used to prepare drug delivery vehicles for effective cancer treatment, serum albumin-based nanovehicles have been widely developed and investigated due to their prominent superiorities, including good biocompatibility, high stability, nontoxicity, non-immunogenicity, easy preparation, and functionalization, allowing them to be promising candidates for cancer diagnosis and therapy. This article reviews the recent advances on the applications of serum albumin-based nanovehicles in cancer diagnosis and therapy. We first introduce the essential information of bovine serum albumin (BSA) and human serum albumin (HSA), and discuss their drug loading strategies. We then discuss the different types of serum albumin-based nanovehicles including albumin nanoparticles, surface-functionalized albumin nanoparticles, and albumin nanocomplexes. Moreover, after briefly discussing the application of serum albumin-based nanovehicles used as the nanoprobes in cancer diagnosis, we also describe the serum albumin-based nanovehicle-assisted cancer theranostics, involving gas therapy, chemodynamic therapy (CDT), phototherapy (PTT/PDT), sonodynamic therapy (SDT), and other therapies as well as cancer imaging. Numerous studies cited in our review show that serum albumin-based nanovehicles possess a great potential in cancer diagnostic and therapeutic applications.

## Introduction

Although tremendous progress has been made by humans in cancer treatment, the number of cancer cases is increasing year by year with the aging of the population and the increase in population, making it the main cause of human death around the world (Bray et al., 2018). The current clinical treatment methods against cancer include surgical resection, chemotherapy, and radiotherapy (Li et al., 2019), and these traditional cancer treatment methods still have many shortcomings. Surgical resection does not completely remove the tumors, which may cause tumor recurrence. For instance, as a sort of highly malignant and invasive brain tumor, glioblastoma has the characteristics of high mortality, poor prognosis, and high recurrence rate. Despite surgery can be utilized to remove glioblastomas, tumor cells that infiltrate the normal brain parenchyma cannot be completely removed by surgery (Xue et al., 2017). Although radiotherapy is a commonly used method for cancer therapy, the tumor resistance induced by hypoxia and the damage to normal tissues caused by high-dose radiation have severely hindered the clinical application of radiotherapy (Huo et al., 2017). Chemotherapy is one of the most common cancer treatments; however, it is easy to cause damage to healthy tissues and organs due to the nonspecific distribution of chemotherapeutics and lack of specificity for tumor cell recognition (Li et al., 2016). In addition, the repeated administration of chemotherapeutic drugs can induce multidrug resistance in tumor cells, which is a major obstacle to the clinical application of chemotherapy (Yang et al., 2018).

Therefore, there is an urgent need for the safe and effective treatment of cancer. Recently, the development of nanotechnology provides a vital platform for cancer therapy, exerts an important influence on the development of versatile drug delivery vehicles, and offers opportunities for controlled drug release and combined cancer treatments (Shi et al., 2010; Shen et al., 2020). A variety of organic or inorganic materials have been used to fabricate the drug delivery vehicles, which have been applied to load diagnostic molecules with imaging functions and therapeutic agents with antitumor effects (Shen et al., 2020). This kind of drug delivery vehicles with dual functions of diagnosis and therapy can realize cancer theranostics (Menon et al., 2013). The drug delivery vehicles have the advantages of large surface area, controllable size, and toilless surface modification, which have been demonstrated to be the smart and multifunctional drug delivery systems that can deliver therapeutic agents into the tumor tissues (Mc Carthy et al., 2015). Proper surface modification (such as PEGylation) or adjustment of the particle size to an appropriate size can evade the phagocytic uptake of drug delivery vehicles by the reticuloendothelial system (RES), resulting in the prolonged circulation time in blood (Huo et al., 2017; Shen et al., 2017). These drug delivery vehicles can passively accumulate in tumor tissues through enhanced permeability and retention effect (EPR effect), and when the targeting ligand or antibody is modified on the surface of drug delivery vehicles, they can specifically recognize and bind to the target cells through the receptor–ligand interactions, thereby realizing the active targeting of tumor regions (Farokhzad and Langer, 2009).

The serum albumin-based nanovehicles is one type of efficient drug delivery vehicles, which utilize serum albumin as the well-behaved carrier materials to encapsulate or conjugate the therapeutic agents for tumor-targeted drug delivery (Elzoghby et al., 2012). Albumin-bound formulation of paclitaxel (Abraxane) is composed of the combination of paclitaxel molecule and human serum albumin through hydrophobic interactions, which has already been approved by the United States Food and Drug Administration (FDA) for clinical treatment of metastatic breast cancer in 2005 (Tomao et al., 2009). Serum albumin, the most abundant protein in plasma, plays a pivotal role in regulating plasma colloidal osmotic pressure and transporting endogenous compounds (An and Zhang, 2017), which possesses prominent superiorities including nontoxicity, non-immunogenicity, good biocompatibility, and high stability, allowing them an ideal candidate for drug delivery vehicle preparation and loading chemotherapy drugs, hypoglycemic drugs, anti-inflammatory drugs, photosensitizers, photothermal agents, and radioisotopes (Elzoghby et al., 2012; Du et al., 2013; Chen et al., 2014; Sheng et al., 2014; Chen et al., 2015; Yao et al., 2019; Yi et al., 2020; Lei et al., 2021). Serum albumin contains both hydrophobic and hydrophilic domains as well as a large number of functional groups, laying a good foundation for its loading of multifarious drugs and functional modification (Bern et al., 2015). Furthermore, the serum albumin is capable of accumulating at tumor tissues or sites of inflammation (Sleep, 2015). The accumulation of serum albumin in tumors is attributed to the efficient interaction of albumin with gp60 receptor (a glycoprotein with molecular weight of 60 kDa), a vascular endothelial membrane protein, and SPARC (secreted protein, acidic and rich in cysteine), an extracellular matrix glycoprotein that is overexpressed in a variety of tumors, which play a pivotal role in the transcytosis of albumin and promote the local concentration of the therapeutic agent-loaded albumin-based nanovehicles in tumors (Kratz, 2008; Bern et al., 2015; Lei et al., 2021).

**FIGURE 1:**
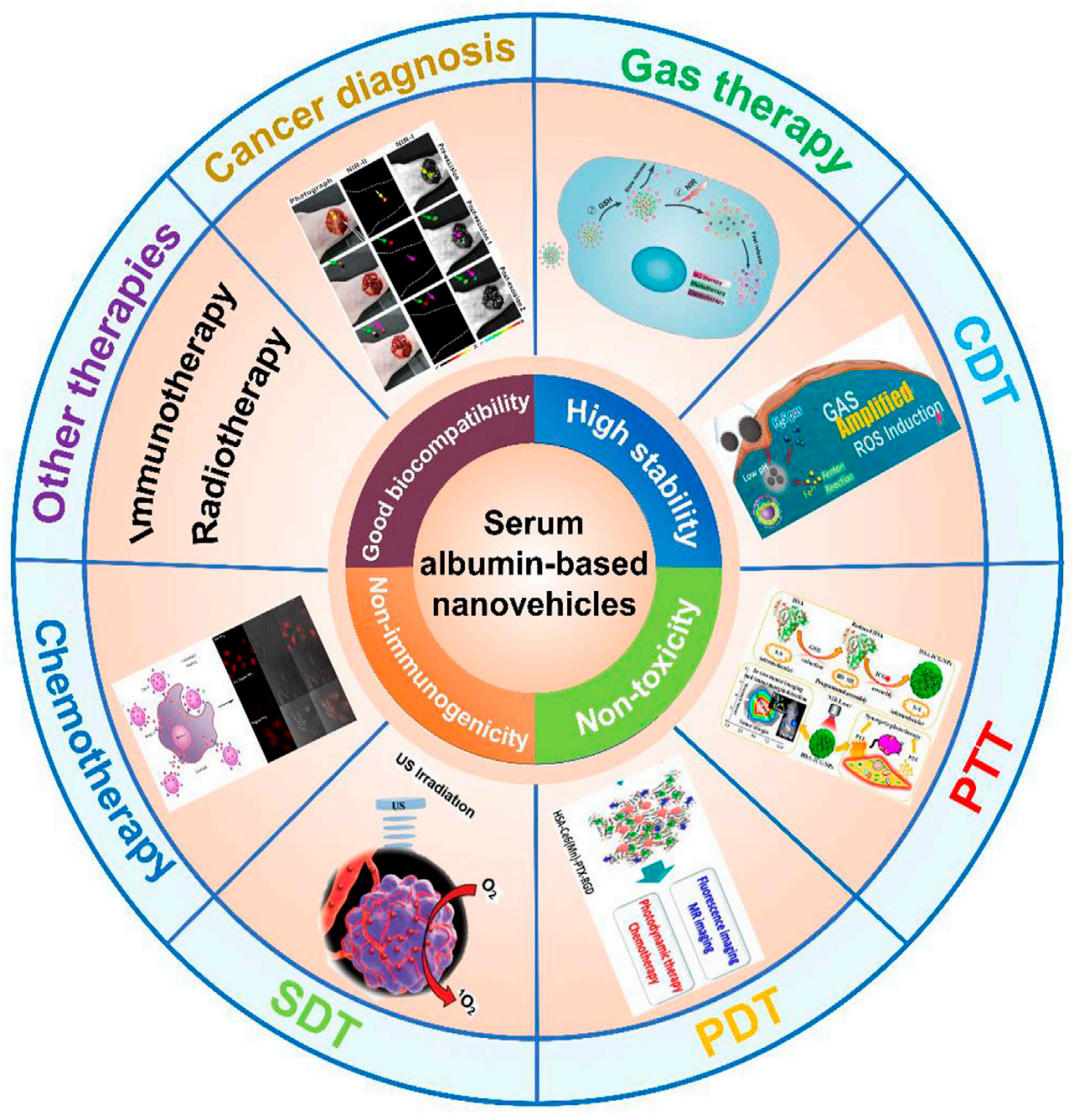
The application of serum albumin-based nanovehicles in cancer diagnosis and therapy. Image for gas therapy: Reproduced with permission from Xu et al. (2019). Copyright 2019, Royal Society of Chemistry. Image for CDT: Reproduced with permission from Xie et al. (2020). Copyright 2020, WILEY-VCH Verlag GmbH and Co. KGaA, Weinheim. Image for PTT: Reproduced with permission from Sheng et al. (2014). Copyright 2014, American Chemical Society. Image for PDT: Reproduced with permission from Chen et al. (2015). Copyright 2015, American Chemical Society. Image for SDT: Reproduced with permission from Ma et al. (2019). Copyright 2018, WILEY-VCH Verlag GmbH and Co. KGaA, Weinheim. Image for chemotherapy: Reproduced with permission from Qi et al. (2015). Copyright 2015, American Chemical Society. Image for cancer diagnosis: Reproduced with permission from Zeng et al. (2020). Copyright 2020, American Chemical Society.

There have been several previously published reviews focusing on the application of albumin-based nanocarriers in drug delivery from different aspects, including preparation methods, surface modification, albumin cellular receptors, albumin-based drug delivery strategies, cancer multimode therapy, drug delivery routes, and market-approved products (Elzoghby et al., 2012; Larsen et al., 2016; Tan and Ho, 2018; Karami et al., 2020; Li et al., 2020). In this review, we highlight the versatility of the serum albumin-based nanovehicles for different applications and briefly summarize the development and clinical potential of the serum albumin-based nanovehicles in cancer diagnosis and therapy. We first describe the essential information of bovine serum albumin (BSA) and human serum albumin (HSA), and discuss their drug loading strategies. Then, we describe the different types of serum albumin-based nanovehicles including albumin nanoparticles, surface-functionalized albumin nanoparticles, and albumin nanocomplexes. Furthermore, after briefly discussing the application of serum albumin-based nanovehicles used as the nanoprobes in cancer diagnosis, we also emphasize the serum albumin-based nanovehicle-assisted cancer theranostics, involving gas therapy, chemodynamic therapy (CDT), phototherapy (PTT/PDT), sonodynamic therapy (SDT), and other therapies as well as cancer imaging. The potential applications of serum albumin-based nanovehicles in cancer diagnosis and therapy are illustrated in Figure 1.

## Drug loading strategies

Albumin is a natural protein, which can be acquired from ovalbumin, human serum albumin (HSA), and bovine serum albumin (BSA), as well as some other sources (Elzoghby et al., 2012; Lei et al., 2021). Among them, HSA and BSA are extensively used in drug delivery vehicle preparation currently and have received widespread attention in the application of cancer diagnosis and therapy. Therefore, this review will center on these two kinds of albumin. The hydrophobic, hydrophilic domains and the large number of functional groups available in the primary structure of serum albumin provide a rational basis for the exploitation of serum albumin for a variety of desired drug delivery (Du et al., 2013; Chen et al., 2014; Chen et al., 2015). HSA is a single-chain polypeptide composed of 585 amino acid residues with a molecular weight of 66,500 Da, containing 17 pairs of disulfide bonds and one sulfhydryl group (Rabbani and Ahn, 2019). HSA is structurally formed by three homologous domains I, II, and III, and each domain contains a pair of helical subdomains A and B (Fasano et al., 2005; Kudarha and Sawant, 2017). There are two significant drug-binding sites named Sudlow’s site I and Sudlow’s site II located in subdomain IIA and subdomain IIIA, respectively, which are responsible for multifarious drug binding (such as chemotherapy drugs, hypoglycemic drugs, and anti-inflammatory drugs, etc.) (Hoogenboezem and Duvall, 2018; Siddiqui et al., 2021). BSA is composed of 583 amino acid residues and shares 75.6% homologous sequence with HSA; therefore, it has a similar structure to that of HSA, which is also capable of binding most drugs (Elzoghby et al., 2012; Lei et al., 2021). After loading the hydrophobic drugs, such as paclitaxel, albumin and the drug molecules will self-assemble into the nanoparticles with the size of 50–150 nm through hydrophobic interaction, leading to the prolonged circulation time, improved targeting property, and reduced side effects of the drugs (Trynda-Lemiesz, 2004; Chen et al., 2015). Several water-soluble drugs such as oligonucleotides and chemotherapy drug doxorubicin are able to be loaded on albumin, forming the nanoparticles by using chemical cross-linking agents (Arnedo et al., 2004; Qi et al., 2015). Moreover, some small-molecule drugs can also be carried on the albumin through the covalent binding or electrostatic adsorption (Stefano et al., 2004; Yu et al., 2016). For instance, methotrexate-conjugated human serum albumin (MTX-HSA) is obtained by directly conjugating the methotrexate (MTX) to the lysine residues of HSA through an amide binding (Burger et al., 2001; Vis et al., 2002; Fiehn et al., 2004). Additionally, the protein-based drugs can be genetically connected to either the C-, N-terminal, or both termini of the albumin to obtain the albumin–protein conjugates, which induce the improved pharmacokinetics (Subramanian et al., 2007; Chen et al., 2010). Besides the ability of loading drugs, albumin is capable of being covalently chemical functionalized and radiolabeled owing to its large number of functional groups (Nosrati et al., 2018; Yi et al., 2020).

## Different types of serum albumin-based nanovehicles

**FIGURE 2:**
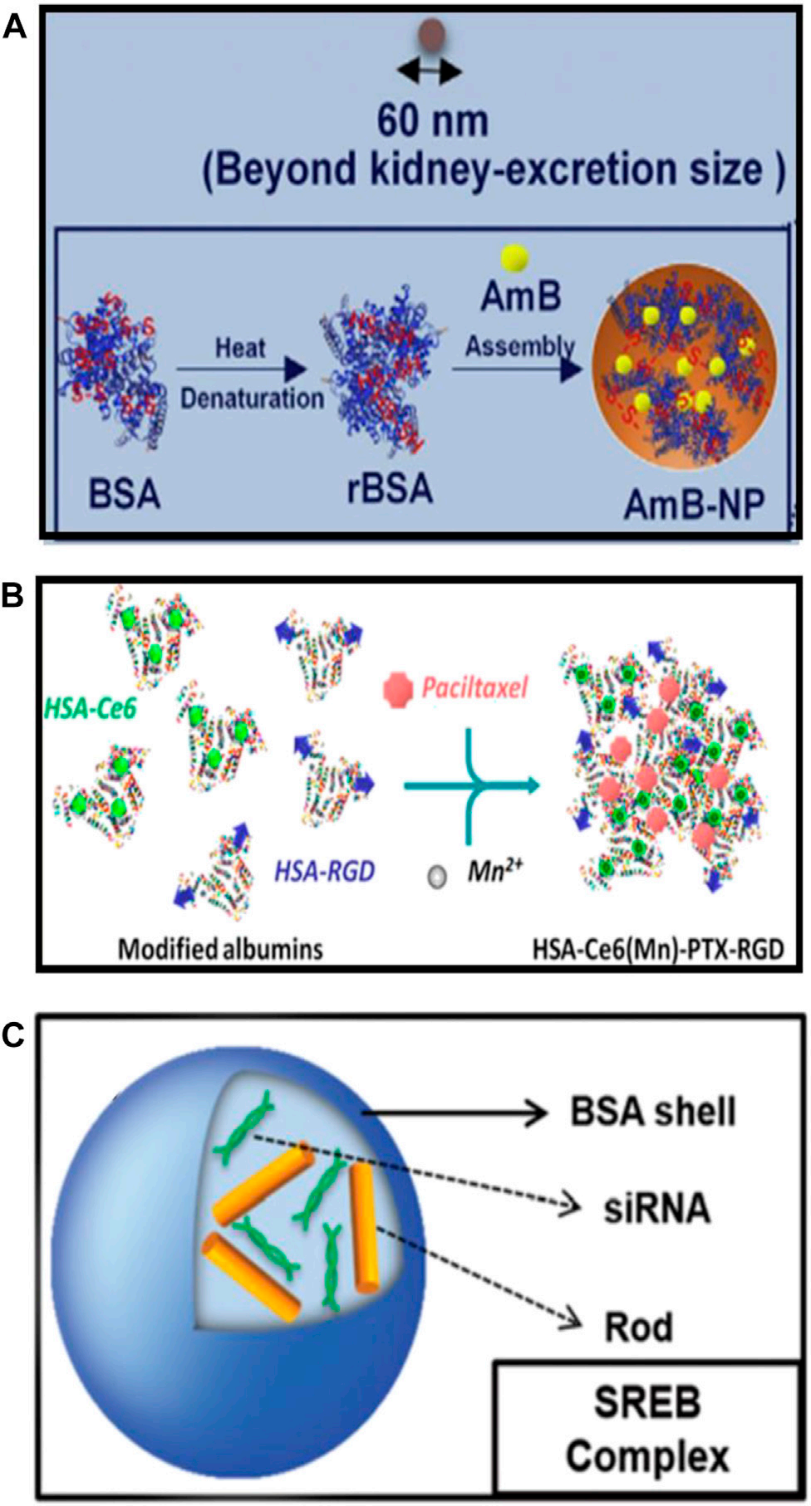
The schematic illustrations of the typical representatives of **(A)** albumin nanoparticles [reproduced with permission from Liu X. et al. (2020); Liu Y. et al. (2020). Copyright 2020, Elsevier], **(B)** surface-functionalized albumin nanoparticles [reproduced with permission from Chen et al. (2015). Copyright 2015, American Chemical Society], and **(C)** albumin nanocomplexes (reproduced with permission from Choi et al. (2015). Copyright 2015, Royal Society of Chemistry).

Different types of serum albumin-based nanovehicles for cancer theranostics involve albumin nanoparticles, surface-functionalized albumin nanoparticles, and albumin nanocomplexes. The variability of the structures of serum albumin-based nanovehicles in terms of composition offers the opportunities for delivery of various cargoes in a controlled and precise manner. The schematic illustrations of the typical representatives of albumin nanoparticles, surface-functionalized albumin nanoparticles, and albumin nanocomplexes are shown in Figure 2.

### Albumin Nanoparticles

As described earlier, serum albumin appears to be capable of loading a variety of therapeutic agents owing to the specific drug-binding sites and abundant functional groups on the albumin. Serum albumin is stable, biocompatible, nontoxic, non-immunogenic, and can accumulate at tumor tissues or sites of inflammation attributing to the interaction of albumin with gp60 receptor and SPARC. Therefore, it is a favorable strategy to use albumin to prepare drug-loaded nanoparticles, which cannot only improve the pharmacokinetics, but also enhance the accumulation of drugs in sites of tumors and inflammation. The techniques of desolvation, emulsification, nanoparticle albumin-bound technology (nab^TM^ technology), and self-assembly are the commonly used methods for preparing albumin nanoparticles and are well known to researchers (Weber et al., 2000; Karami et al., 2020; Van de Sande et al., 2020). Sheng et al. (2014), in their 2014 ACS Nano article, employed an assembly strategy to prepare the indocyanine green (ICG)-loaded HSA nanoparticles (HSA-ICG NPs) by intermolecular disulfide conjugations. The disulfide bonds of HSA molecular were cleaved by incubating the HSA and ICG with excessive glutathione (GSH), then the resulting free sulfhydryl groups were assembled again through intermolecular disulfide bonds to form HSA–ICG NPs with a hydrodynamic diameter of around 75 nm and 11% loading rate of ICG. Park et al. (2021) have designed the PEGylated nanoparticle albumin-bound steroidal ginsenoside to alleviate SARS-CoV-2-mediated hyper-inflammatory responses and demonstrated its potential applications in the treatment of symptoms including coagulation and cytokine storm in severe SARS-CoV-2 patients. Kalashnikova et al. (2020) have synthesized the albumin-cerium oxide nanoparticles with indocyanine green (ICG) dye conjugation, which were proven to be accumulated in inflamed joints and showed a similar therapeutic effect to that of methotrexate against rheumatoid arthritis. Amphotericin B (AmB) is a potent fungicide that can bind to albumin *in vivo* to form a nanocomplex smaller than 10 nm, which is easy to accumulate in the kidneys, and then interacts with cholesterol on renal epithelial cells, eventually leading to the nephrotoxicity (Hamill, 2013; Tang et al., 2015; Gurudevan et al., 2018). Liu et al. took advantage of the interaction between AmB and albumin to directly assemble BSA and AmB into larger-sized nanoparticles, which could evade kidney excretion (Figure 2A). The obtained AmB-bovine serum albumin (BSA) nanoparticles (AmB-NP) exhibited the efficient AmB loading and reshaped the biodistribution of AmB *in vivo*, resulting in reduced nephrotoxicity and improved antifungal activity (Liu Y. et al., 2020).

### Surface-Functionalized Albumin Nanoparticles

Although the EPR effect and the interaction of albumin with gp60 receptor and SPARC can enhance the accumulation of drug-loaded albumin nanoparticles in tumors, they still have some drawbacks, such as insufficient tumor targeting and weak tumor tissue penetration. Modification of ligands on the surface of albumin nanoparticles that can bind to the specific receptors on the surface of target cells is able to improve the targeting ability, pharmacokinetics, and biodistribution of the drugs, resulting in the enhanced therapeutic effects and reduced side effects. Chen et al. have fabricated the photosensitizer chlorin e6 (Ce6), and the chemotherapy drug paclitaxel (PTX) co-loaded human serum albumin (HSA) nanoparticles with cRGDyK peptide modification (Figure 2B), which were able to target tumor angiogenic endothelium overexpressing α_v_β_3_-integrin and exhibited significant antitumor efficacy due to the combined photodynamic/chemotherapy (Chen et al., 2015). Ma et al. (2020) have presented an antiplatelet strategy based on erythrocyte membrane-coated bovine serum albumin (BSA) nanoparticles that co-load L-Arginine (LA) and photosensitizer IR783. They first used BSA as the scaffold to co-load LA and IR783, then the obtained drug-loading albumin nanoparticles were coated with red blood cell (RBC) membrane, and finally, the cRGD peptides were integrated into the lipid bilayer of RBC membrane to get the cRGD peptides conjugated and erythrocyte membrane-coated BSA nanoparticles with co-loading of LA and IR783. The results indicated that the as-designed surface-functionalized albumin nanoparticles can actively target the integrin receptor (integrin α_IIb_β_3_, α_v_β_3_) overexpressed platelets and tumor cells, respectively, facilitating the inhibition of platelet activation and cellular uptake of nanoparticles by tumors.

### Albumin Nanocomplexes

Albumin nanocomplexes are formed of albumin with gene, lipid, polymer, and some other materials (Rhaese et al., 2003; Xu et al., 2010; Choi et al., 2015; Zhu et al., 2017; Yu et al., 2020). The novel bovine serum albumin (BSA)-based nanocomplexes encapsulated with both Bcl-2-specific small interfering RNA (siRNA) and gold (Au) nanorods were developed (Figure 2C), and the downregulation efficacy of siRNA on Bcl-2 antiapoptotic protein expression as well as the photothermal property of nanocomplexes were further estimated with the aim of providing an efficient approach for targeted breast cancer treatment (Choi et al., 2015). These albumin nanocomplexes exerted remarkable anticancer efficacy through the combined photothermal therapy and siRNA-mediated gene silencing, leading to around 80% of cancer cell death *in vitro*. Although the described multicomponent albumin nanocomplexes have a relatively larger size than the clinical used diameter (about <200 nm), their biocompatibility and synergistic antitumor effects prevail over the previous lipid-based and polymer-based nanocomplexes, which show a great potential for the further application in synergistic cancer gene and photothermal therapy *in vivo* (Choi et al., 2015). Although the small-sized albumin nanocomplexes (less than 10 nm) may be conducive to the tumor penetration after i.v. administration, nanocomplexes with such a small particle size showed insufficient circulation time in blood and poor tumor accumulation (Taguchi et al., 2012; Huo et al., 2017), resulting in a compromised therapeutic effect. Therefore, Yu et al. (2020) used FAP-α-responsive cleavable amphiphilic peptide-modified thermosensitive liposomes to encapsulate small-sized albumin–paclitaxel nanoparticles (HSA-PTX); then a photothermal agent IR-780 was incorporated into the lipid bilayer of thermosensitive liposomes to obtain the FAP-α and hyperthermia-responsive lipid–albumin nanocomplexes. These nanocomplexes were validated to be able to accumulate at tumor regions and release HSA-PTX triggered by FAP-α that specifically expresses on cancer-associated fibroblasts (CAFs), then the release of small-sized HSA-PTX was enhanced for deep tumor penetration and tumor killing through the produced hyperthermia upon near-infrared (NIR) laser irradiation, suggesting that the encapsulation of small-sized HSA-PTX into thermosensitive liposomes can overcome the disadvantages of small nanoparticles including short circulation time and poor tumor accumulation.

## Serum albumin-based nanovehicles as nanoprobes for cancer diagnosis

**FIGURE 3:**
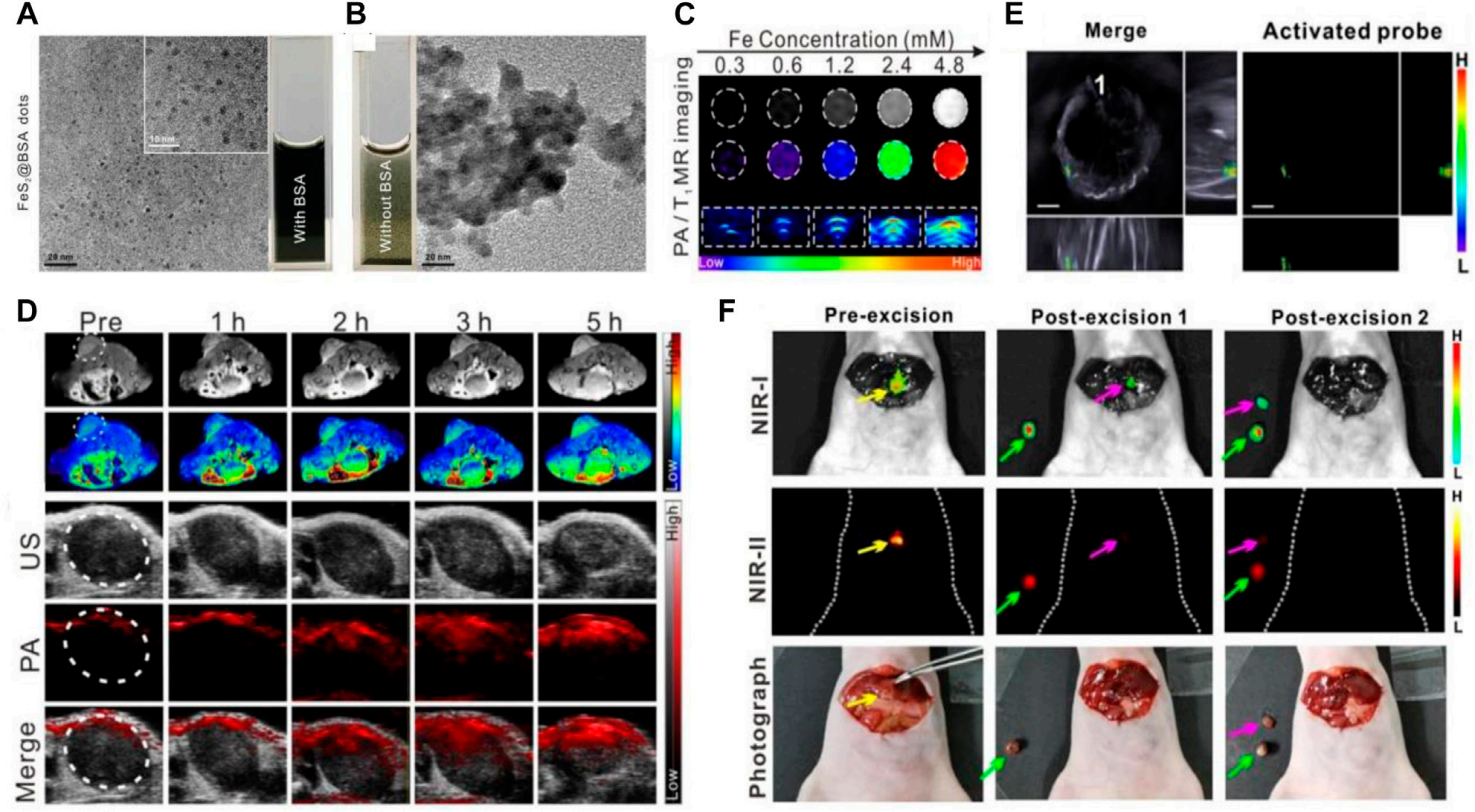
Serum albumin-based nanovehicles as nanoprobes for cancer diagnosis. **(A)** TEM image of the synthesized FeS@BSA QDs. **(B)** TEM image of FeS synthesized without BSA. **(C)** *In vitro* T_1_-weighted magnetic resonance (MR) images and photoacoustic (PA) images of FeS@BSA QDs at various concentrations. **(D)** Time-dependent T_1_-weighted MR images, PA, and ultrasound (US) images of tumor before and after i.v. administration of FeS@BSA QDs. Reproduced with permission from Yang et al. (2020). Copyright 2020, Elsevier. **(E)** The 3D MSOT images of orthotopic liver tumor upon i.v. administration of BH-NO_2_@BSA. **(F)** The NIR-I/NIR-II fluorescence images and white-light photographs of a mouse with exposed liver and orthotopic liver tumor at the state of pre-excision, post-excision 1, and post-excision 2. The yellow, green, and pink arrows point to the orthotopic liver tumor, the excised tumor, and the excised residual tumor, respectively. Reproduced with permission from Zeng et al. (2020). Copyright 2020, American Chemical Society.

Recently, serum albumin-based nanovehicles have been extensively utilized in the diagnosis of diseases owing to their significant superiorities mentioned above. ^99m^Tc aggregated albumin, a sterile injectable radiopharmaceutical, which has been developed for various clinical diagnostic applications, including the detection of sentinel node in breast cancer and diagnosis of other solid tumors (Rink et al., 2001; Dhabuwala et al., 2005; Weiss et al., 2005). Zhang et al. (2019) have designed a newfangled IR820–maleimide conjugate (IR-Mal) that can covalently bind the tissue interstitial albumin *in vivo* for sentinel lymph node (SLN) dual-mode imaging. The IR-Mal conjugate showed bright blue appearance so it can be used for the naked-eye SLN identification. After covalently binding to the tissue interstitial albumin *in vivo*, the obtained BSA–IR–Mal complexes showed a remarkably stronger fluorescence intensity than that of IR-Mal and have been corroborated to have the ability to specifically accumulate in lymph nodes and detect both normal and metastatic SLN through naked-eye identification and high-resolution fluorescence imaging. Yang et al. have fabricated the extremely small-sized FeS QDs by employing an albumin-constrained strategy without using any complicated chemical synthesis procedures, which showed excellent longitudinal relaxivity and strong absorption at the near-infrared region. The TEM image in Figure 3A indicated that the synthesized FeS@BSA QDs were well dispersed and had uniform and ultrasmall particle size of approximate 3 nm. Contrarily, the large-sized aggregates were found when BSA was not used to constrain FeS (Figure 3B). BSA was discovered to play a vital role in the synthesis process and was used as a carrier for multifunctionality, which constrains the overgrowth of particles in the reaction and prevents the particles from aggregation. The T_1_-weighted magnetic resonance imaging (MRI) and photoacoustic (PA) imaging capability of FeS@BSA QDs were further evaluated, and the MR and PA imaging signals were found to increase in a gradient concentration-dependent manner *in vitro* (Figure 3C). The T_1_-weighted MRI images showed that FeS@BSA QDs could remarkably illuminate the tumor area (Figure 3D), and the positive MRI signal intensity at the tumor site was gradually increased after injection with FeS@BSA QDs solution through the tail vein, and the signal was magnified by 1.8-fold at 5 h post-injection. The tumor region in tumor-bearing mouse after i.v. administration of FeS@BSA QDs was also imaged under PA mode and showed a gradually intensive PA signal intensity with an increase in time after FeS@BSA QDs injection (Figure 3D). The *in vivo* and *in vitro* imaging results not only indicated the ability of FeS@BSA QDs to accumulate in the tumor region but also suggested their inherent MR and PA imaging capabilities at tumor sites. This work demonstrated the tremendous clinical translational potential in T_1_-weighted MRI of diseases and inspired the application of other functional nanoprobes in tumor diagnosis (Yang et al., 2020). The accurate localization and complete excision of tumors are pivotal to the successful cancer surgical treatment (Keereweer et al., 2013; Chi et al., 2014; Ou-Yang et al., 2019). Thus, Zeng et al. (2020) have designed and synthesized the nanocomposite probe (BH-NO_2_@BSA), which consists of a molecular probe (BH-NO_2_) and the carrier BSA, which can specifically respond to nitroreductase overexpressed in tumors, and then the aromatic nitro group transforms into an electron-donating amino group and finally activate the nanoprobe. The activated nanoprobe showed an intensive absorption in the near-infrared (NIR) region and was capable of generating NIR-I/II fluorescence signals at about 791 nm (excited at 680 nm) or 923 nm (excited at 808 nm), which endowed the albumin-based nanoprobes with the capability of multispectral optoacoustic tomography (MSOT) and NIR-I/NIR-II fluorescence imaging of tumors for preoperative tumor location and intraoperative tumor surgery navigation. The 3D MSOT images before surgical excision of tumors were obtained to locate the orthotopic liver tumor upon i.v. administration of BH-NO_2_@BSA (Figure 3E). Then the nanoprobe solution was sprayed onto the liver of the mice after the skin and peritoneum of the mouse were removed in order to observe the clear fluorescence signal boundaries in the tumor site (pre-excision) owing to the activation of the nanoprobe by the overexpressed nitroreductase. The orthotopic liver tumor was excised (post-excision 1) after being accurately located and was laid next to the mouse body. Subsequently, the residual tumor tissue was identified and removed (post-excision 2) in the intraoperative guidance of the NIR-I and NIR-II fluorescence imaging and was placed next to the mouse body (Figure 3F). Compared with the enhanced fluorescence signals in the NIR-I/NIR-II fluorescence images, the residual tumor tissue in the site where the orthotopic tumor was excised in white-light photograph (post-excision 1) was not easily distinguished from the surrounding normal tissue. The nanoprobe BH-NO_2_@BSA was confirmed to hold a great potential for preoperative tumor location and intraoperative tumor surgery navigation through potent MSOT and NIR-I/NIR-II fluorescence imaging, respectively.

## Serum albumin-based nanovehicle-assisted cancer theranostics

In recent years, the nanomedicine-based novel tumor treatment approaches, including photodynamic therapy, photothermal therapy, immunotherapy, etc., have been continuously developed and researched with the rapid development of nanotechnology. However, most of these approaches are still in the stage of basic research or clinical trials, and face a series of challenges and problems. The accumulation of nanomedicine in tumors is not only affected by the passive targeting effect but also closely related to the active targeting functional group of nanomedicine (Bertrand et al., 2014; Wang et al., 2016). Moreover, the type and size of tumors also significantly affect the accumulation of nanomedicine at the tumor site and the therapeutic effect (Zhan et al., 2017). Therefore, *in situ* and real-time monitoring of the absorption, distribution, metabolism, and excretion processes of nanomedicine *in vivo*, as well as timely evaluation of the therapeutic effect, have become a pivotal issue for nanomedicine in cancer treatment (Shen et al., 2020). The “theranostics” was first proposed by John Funkhouser in 1998 (Lim et al., 2015), and it is now generally accepted that “theranostics” is a new type of biomedical technology that combines disease diagnosis or monitoring with treatment (Lim et al., 2015; Chen et al., 2017). Nowadays, researchers have developed various drug delivery vehicles, which co-load diagnostic molecules and therapeutic agents and, thus, can realize cancer theranostics, bringing new hope for humans to overcome cancer. The serum albumin-based nanovehicle-assisted cancer theranostics, involving gas therapy, chemodynamic therapy (CDT), phototherapy (PTT/PDT), sonodynamic therapy (SDT), and other therapies as well as cancer imaging will be briefly summarized as follows. Representative applications of serum albumin-based nanovehicles for cancer theranostics are listed in Table 1.

**TABLE 1:** Summary of representative applications of serum albumin-based nanovehicles for cancer theranostics.

Therapy	Formulation	Nanovehicle type	Therapeutic agent	Imaging modality	Administration route	Applications	Ref.
Gas therapy	IPH-NO	Albumin nanoparticles	NO, PTX, IR780	Fluorescence imaging	Intravenous administration	Breast cancer treatment (4T1 cells)	Xu et al. (2019)
CDT	FeS@BSA	Albumin nanoparticles	Fe^2+^, H_2_S	T_2_-weighted MRI	Intravenous administration	Hepatoma carcinoma treatment (Huh7 cells)	Xie et al. (2020)
PTT	HSA-ICG	Albumin nanoparticles	ICG	NIR fluorescence imaging, PA imaging	Intravenous administration	Breast cancer treatment (4T1 cells)	Sheng et al. (2014)
PDT	HSA-Ce6(Mn)-PTX–RGD	Surface-functionalized albumin nanoparticles	Ce6, PTX	T_1_-weighted MRI, fluorescence imaging	Intravenous administration	Human glioblastoma treatment (U87MG cells)	Chen et al. (2015)
SDT	MTTP–HSA	Albumin nanocomplexes	metal 4-methylphenylporphyrin (TTP) complexes	T_1_-weighted MRI, PA imaging	Intravenous administration	Breast cancer treatment (MCF-7 cells)	Ma et al. (2019)
Chemotherapy	DOX/GA-rHSA	Albumin nanoparticles	DOX	Fluorescence Imaging	Intravenous administration	Liver cancer treatment (H22 liver tumors, HepG 2 cells)	Qi et al. (2015)
Immunotherapy	Al-BSA-Ce6	Albumin nanoparticles	Ce6, aluminum adjuvant	Fluorescence imaging	Intravenous administration	Melanoma treatment (B16F10 cells)	Zhu et al. (2020)
Radiotherapy	^125^I-HSA/^131^I-HSA	Albumin nanoparticles	^125^I/^131^I	SPECT/CT imaging	Intravenous administration	Colon cancer treatment (CT26 cells)	Yi et al. (2020)

### Gas Therapy-Based Cancer Theranostics

**FIGURE 4:**
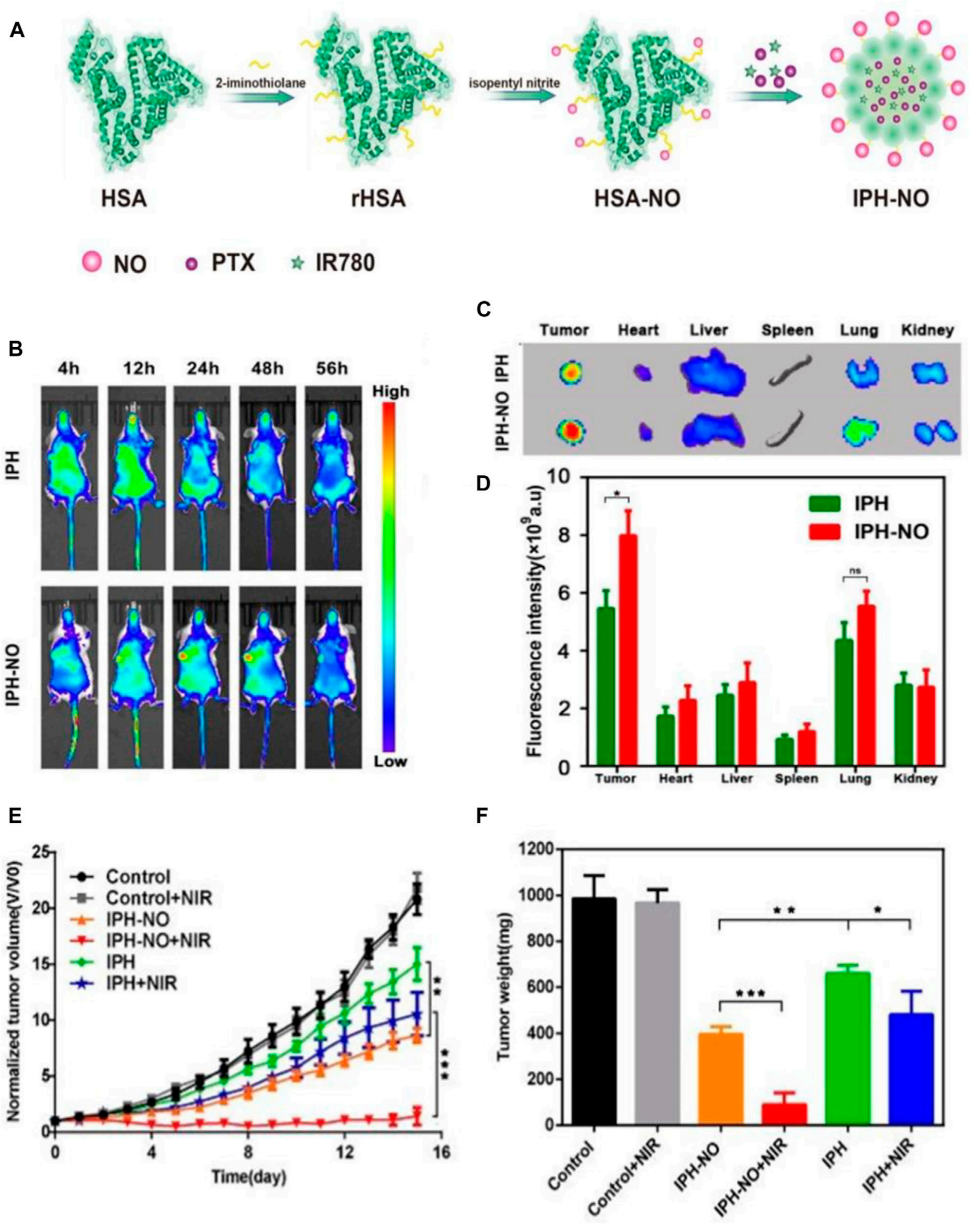
Gas therapy-based cancer theranostics. **(A)** Schematic illustration of the synthesis procedure of IPH–NO. **(B)** The whole-body fluorescence images of 4T1 tumor-bearing mice at different time points after i.v. administration of IPH or IPH-NO. **(C)** Fluorescence images of the excised tumors and major organs from indicated mice at 56 h post-injection. **(D)** Quantification of fluorescence signals of the isolated organs and tumors. **(E)** Tumor growth curves in mice from different treatment groups. **(F)** Weight of the excised tumors in different treatment groups at day 15. *p < 0.05, **p < 0.01, ***p < 0.001. Reproduced with permission from Xu et al. (2019). Copyright 2019, Royal Society of Chemistry.

Drug delivery vehicle-based cancer theranostics have been demonstrated to be a promising strategy for cancer treatment owing to their ability to accumulate in solid tumors via the EPR effect, which provides an approach for drug delivery vehicles to enter the tumor tissues through the interstice between vascular endothelial cells and remain entrapped in the tumor tissues (Fang et al., 2011; Shen et al., 2017; Shen et al., 2019). However, previous studies have shown that the average tumor uptake of drug delivery vehicles is less than 1% of the injected dose, leading to the unsatisfactory therapeutic effect, which may arise from the tumor vascular barrier obstructing the competent perfusion and retention of drug delivery vehicles in tumors (Wilhelm et al., 2016). Therefore, a strategy that can break the tumor vascular barrier and enhance tumor vascular permeability to improve the therapeutic effect is imminently needed. Xu and coworkers designed an NO donor-S-nitrosated HSA (HSA–NO) to co-load near-infrared (NIR) light-absorbing agents (IR780) and the chemotherapy drug PTX for GSH and NIR-responsive NO release, which can enhance the tumor vascular permeability at low NO concentrations and directly kill cancer cells at high NO concentrations. First, HSA was incubated with isopentyl nitrite to synthesize HSA–NO, and then IR780 and PTX were loaded onto HSA–NO to obtain the codelivery system IPH–NO (Figure 4A). *In vitro* studies demonstrated that the reductive tumor environment can trigger the slow release behavior of NO, and NIR irradiation can induce the quick release behavior of NO to reach high concentrations for tumor growth inhibition. The *in vivo* drug distribution assay was performed to confirm whether NO released from IPH–NO can enhance the tumor vascular permeability and, thus, increase the accumulation of drug at the tumor region. The whole-body fluorescence imaging of 4T1 tumor-bearing mice at different time points after i.v. administration of IPH or IPH–NO (IR780 1.5 mg/ ml) was carried out (Figure 4B). The IPH–NO group displayed a considerable fluorescence signals of IR780 in the tumor region at 24 h post-injection when compared with the IPH group. The excised tumor in the IPH–NO group also showed the intensive fluorescence signals at 56 h post-injection, which showed a 1.46-fold higher fluorescence intensity than that of the IPH group (Figures 4C, D). These results demonstrated the good ability of IPH–NO to accumulate in tumor tissues. The *in vivo* antitumor assay demonstrated that the IPH–NO-treated mice upon NIR irradiation showed the slowest tumor growth rate (Figure 4E) and had the lowest tumor weight among all groups (Figure 4F). The slowly released NO promoted the accumulation of nanoparticles in tumors, leading to the improved chemotherapy efficacy. Then the NIR irradiation triggered the quick release of NO to reach a high NO concentration for NO gas therapy and, combined with the phototherapy of IR780 activated by NIR, significantly inhibited tumor growth (Xu et al., 2019). Another group reported a cRGD-immobolized erythrocyte membrane-enveloped BSA nanoparticle to co-load L-arginine (LA) and photosensitizer IR783 for local NO release, which was able to specifically inhibit the activation of tumor-associated platelets and thereby enhance the vascular permeability and promote the accumulation of the nanoparticles in tumors. The *in vivo* fluorescence imaging and antitumor assays validated their ability to actively target platelets and cancer cells as well as significantly inhibit tumor growth (Ma et al., 2020).

### Chemodynamic Therapy-Based Cancer Theranostics

**FIGURE 5:**
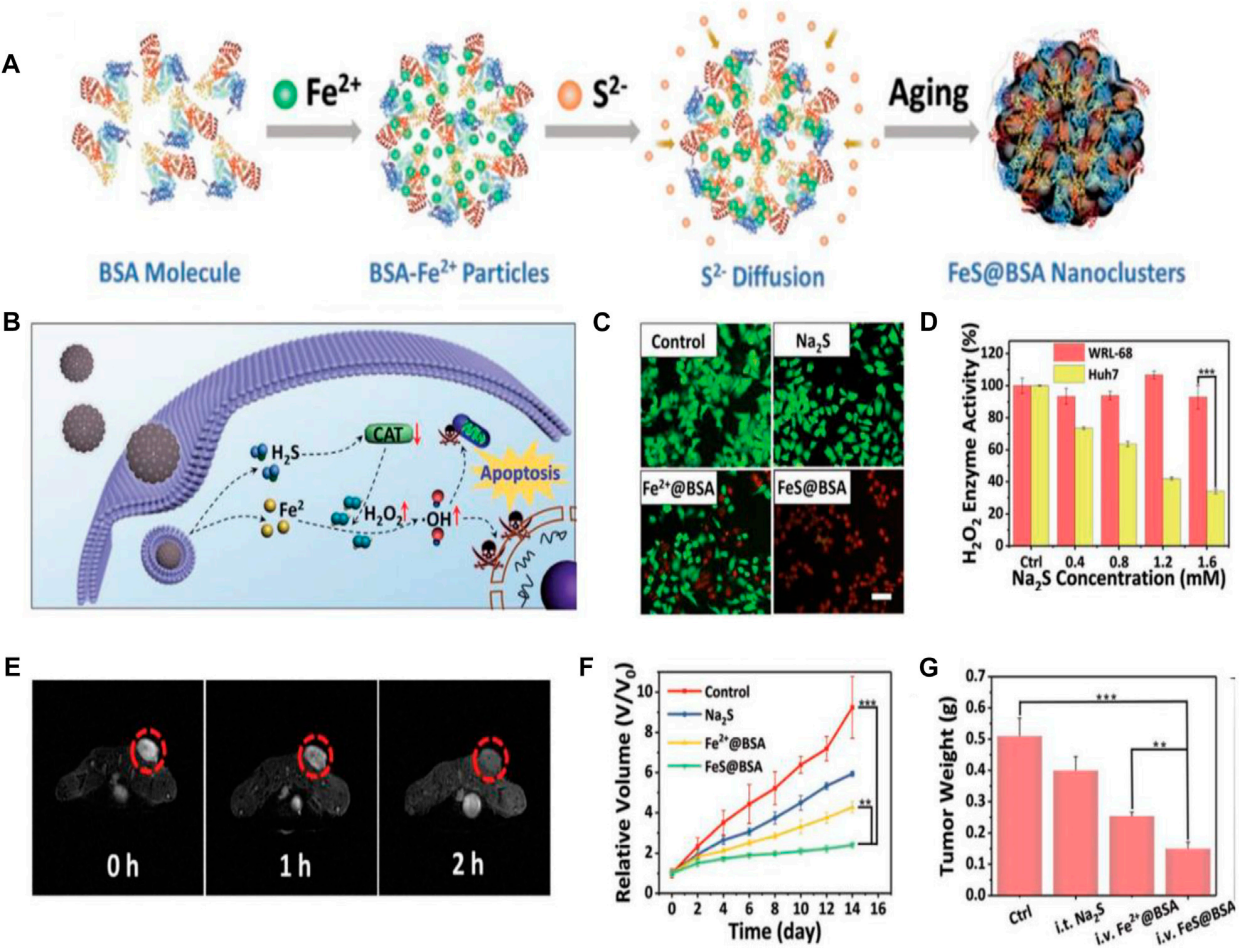
Chemodynamic therapy (CDT)-based cancer theranostics. **(A)** Schematic illustration of the synthesis procedure of FeS@BSA nanoclusters. **(B)** Schematic illustration of synergistic antitumor mechanism of FeS@BSA nanoclusters. **(C)** LIVE/DEAD cell staining of Huh7 cells with calcein-AM/propidium iodide after incubation with PBS, Na_2_S, Fe^2+^@BSA, and FeS@BSA for 24 h. Scale bar is 20 μm. **(D)** Suppression effect of H_2_S on intracellular H_2_O_2_ enzyme activity in WRL-68 and Huh7 cells after incubation with Na_2_S (H_2_S donor). **(E)** T_2_-weighted MR images of Huh7 tumor-bearing nude mice before and after i.v. injection of FeS@BSA. **(F)** Tumor growth curves in mice from different treatment groups. **(G)** Weight of the excised tumors in different treatment groups after the 14 days of treatment. *p < 0.05, **p < 0.01, ***p < 0.001. Reproduced with permission from Xie et al. (2020). Copyright 2020, WILEY-VCH Verlag GmbH and Co. KGaA, Weinheim.

Reactive oxygen species (ROS) can damage biomolecules, such as lipids, proteins, and DNA, and consequently induce cell apoptosis (Wang et al., 2019). Recent efforts have been devoted to the development of cancer treatment strategies based on ROS. In particular, chemodynamic therapy (CDT) has been proven to hold the tremendous potential in cancer treatment (Tang et al., 2017). For example, iron-based CDT has been confirmed to induce tumor cell apoptosis by converting endogenous H_2_O_2_ into cytotoxic hydroxyl radical (•OH) via iron-mediated Fenton reaction (Liu X. et al., 2020). However, the intracellular content of H_2_O_2_ is not sufficient to maintain the continuous production of hydroxyl radical (•OH), which limits the therapeutic effect of CDT (Bechtel and Bauer, 2009). To achieve the enhanced CDT efficacy, Xie and coworkers designed the ferrous sulfide-embedded BSA (FeS@BSA) nanoclusters by diffusing the Fe^2+^ and S^2−^ ions into BSA nanoparticles via a self-assembly approach for H_2_S-amplified CDT of tumors (Figures 5A, B). The employment of BSA as the carrier matrix not only ensures the stability and biocompatibility of the drug delivery vehicles but also serves as a protective agent for FeS compounds from oxidation in the physiological environment. The results demonstrated the capability of FeS@BSA nanoclusters to induce the rapid Fenton reaction to produce hydroxyl radical (•OH) and release H_2_S effectively within Huh7 cancer cells. The LIVE/DEAD cell staining assay confirmed that FeS@BSA nanoclusters can significantly kill cancer cells when compared with other groups (Figure 5C), arising from the intracellular corelease of Fe^2+^ and H_2_S. This result also revealed that H_2_S itself did not induce cell apoptosis directly, but clearly enhanced the CDT triggered by iron-mediated Fenton reaction. The H_2_S-amplified CDT was proven to be related to the inhibition effect of H_2_S molecules on catalase (CAT) activities. CAT can decompose H_2_O_2_ and reduce the production of cytotoxic hydroxyl radical (•OH), consequently leading to the compromised CDT (Bechtel and Bauer, 2009). Figure 5D revealed the effective suppression effect of H_2_S on intracellular H_2_O_2_ enzyme activity in Huh7 cells, which incubated with Na_2_S (H_2_S donor), while the suppression effect of H_2_S on catalase activity in normal cells was negligible. The T_2_-weighted negative MRI was performed by i.v. injection of FeS@BSA in Huh7 tumor-bearing nude mice, and the MR images were obtained at 0 (pre-injection), 1, and 2 h post-injection, which showed the decreased T_2_-weighted negative MRI signals (signal darkening) in the tumor site after the injection, suggesting the enhanced accumulation of FeS@BSA nanoclusters in tumor tissue (Figure 5E). After 14 days of treatment, mice i.v. injected with FeS@BSA showed a slow tumor growth curve and the tumor growth inhibition rate reached approximately 71 wt%, when compared with the approximately 27 and 50 wt% of tumor growth inhibition rates, respectively, in Na_2_S- and Fe^2+^@BSA-treated groups (Figures 5F, G). The H_2_S-amplified CDT for tumors was mainly ascribed to the synergetic effect of Fe^2+^ and H_2_S. These findings corroborated the tremendous potential of FeS@BSA nanoclusters in gas-amplified CDT for cancers with MRI guidance (Xie et al., 2020). In another study, researchers used BSA as a template to synthesize the biodegradable, size-controllable, and metastable γ-phase MnS; the obtained MnS@BSA was able to produce H_2_S and release Mn^2+^ for Fenton-like reaction to generate •OH in the acidic microenvironment of the tumor. The *in vitro* and *vivo* results also demonstrated the ability of MnS@BSA to monitor the cancer treatment process by using as an MRI contrast agent and revealed that MnS@BSA could be used as a theranostic agent for T_1_-weighted MRI, which guided the combination of gas therapy and CDT (He et al., 2020). Liang et al. developed an albumin-based nanoagent, in which manganese oxide and copper oxide are grown *in situ* within the BSA molecules through a biomineralization process, and the Pt (IV) prodrug is conjugated with BSA molecules through an amide bond. The final obtained albumin-based nanoagent can consume GSH through the reaction of manganese oxide with GSH, leading to GSH depletion, while the produced Mn^2+^ and Cu^2+^ can undergo a Fenton-like reaction to convert H_2_O_2_ into •OH, resulting in GSH depletion-enhanced CDT and chemotherapy. Moreover, the released Mn^2+^ enhanced the contrast of MRI, realizing MRI-monitored CDT and chemotherapy (Liang et al., 2021).

### Phototherapy-Based Cancer Theranostics

**FIGURE 6:**
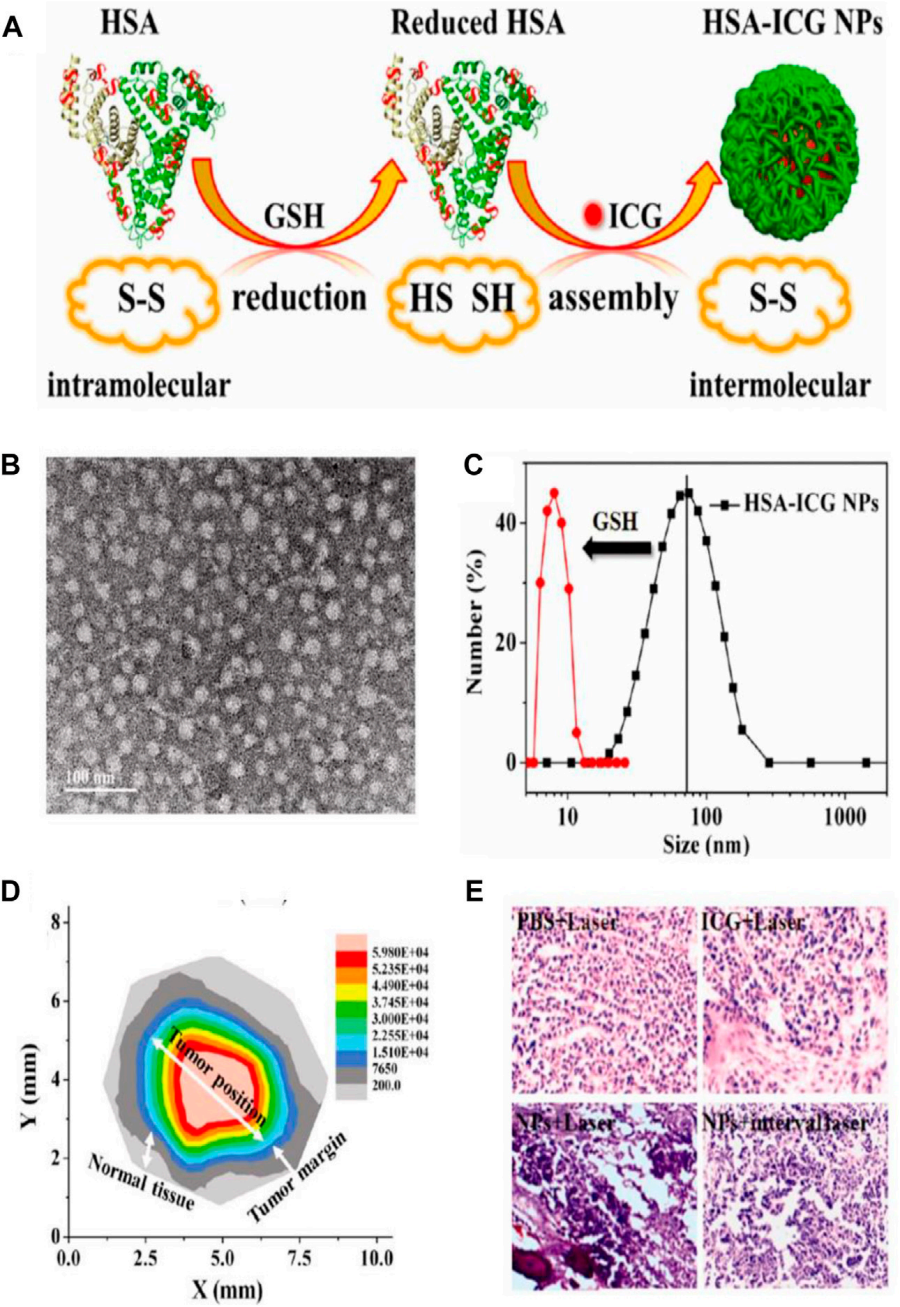
Phototherapy-based cancer theranostics. **(A)** Schematic illustration of the synthesis procedure of human serum albumin (HSA)–indocyanine green (ICG) NPs. **(B)** TEM image of HSA–ICG NPs. **(C)** Size distribution of HSA-ICG NPs measured by DLS. **(D)** Tumor mapping imaging with integrated FL intensity of HSA–ICG NPs at different locations. **(E)** The hematoxylin and eosin (H and E)-stained images of tumor sections collected from different treated groups (PBS + Laser; ICG + Laser; NPs + Laser; NPs + interval Laser) of mice at 4 h post-injection via tail vein. Reproduced with permission from Sheng et al. (2014). Copyright 2014, American Chemical Society.

Nowadays, NIR-light-absorbing agents have been proven to readily convert light energy into thermal energy and/or generate reactive oxygen species (ROS) after exposure to NIR laser irradiation by placing the laser probe on tumor region, achieving photothermal therapy (PTT) and/or photodynamic therapy (PDT) for cancers (Shen et al., 2019). The wavelength range of NIR light is 650–950 nm, and the skin absorbs little light in this NIR window, resulting in low phototoxicity to the skin (Yu et al., 2016). Therefore, the NIR-light-induced PTT or PDT can be used as a minimally invasive approach for tumor treatment. Although multitudinous NIR-light-absorbing agents have been validated to be available for cancer therapy and show encouraging therapeutic effects, they still possess some drawbacks. Fox example, an NIR cyanine dye, indocyanine green (ICG), which has been approved by the FDA, is unstable in aqueous solutions and easily self-bleach and is rapidly eliminated from the body, leading to their poor photostability and unfavorable antitumor effect (Ma et al., 2013; Shen et al., 2019; Chen et al., 2020). To cope with these issues, various nanocarriers, such as liposomes (Lajunen et al., 2016), micelles (Kirchherr et al., 2009), and vesicles (Zhu et al., 2015), have been developed to effectively deliver ICG for improving stability and tumor targeting. Compared with the exogenous nanocarriers, using endogenous HSA as the delivery vehicle is of immense benefit to deliver ICG. First, HSA-based ICG delivery vehicles are biocompatible, nontoxic, non-immunogenic, and stable. Then, HSA-based ICG delivery vehicles can realize tumor-targeted delivery through EPR effect and gp60 and SPARC receptor-mediated transcytosis. Therefore, Sheng and coworkers used endogenous HSA as a delivery vehicle to prepare the ICG-loaded HSA nanoparticles (HSA-ICG NPs) by intermolecular disulfide conjugations through an assembly strategy. First, the HSA and ICG were incubated with excessive glutathione (GSH) to cleave the disulfide bonds of the HSA molecule; then the resulting free sulfhydryl groups were assembled again through intermolecular disulfide bonds to form HSA–ICG NPs (Figure 6A). TEM image revealed that the HSA–ICG NPs were well dispersed and spherical, with the size ranging from 25 to 35 nm (Figure 6B). The average hydrodynamic size of the formed HSA–ICG NPs was around 75 nm measured by DLS, which was larger than the average diameter measured by TEM. It was noted that the average hydrodynamic size of HSA-ICG NPs was decreased to around 8.0 nm when adding GSH (50 mM) into the HSA–ICG NP solution (Figure 6C), suggesting the prominent reductive-sensitive activity of HSA–ICG NPs. The HSA–ICG NPs were also demonstrated to show the excellent property of NIR fluorescence (FL) and PA imaging. Although the real-time NIR FL and PA dual-modal imaging is useful in determining tumor location, the scattering of light in tumor tissues severely hinders the imaging of tumor margin (Sheng et al., 2013; Liang et al., 2014). Therefore, the tumor margin detection was further investigated by using spectrum-resolved technology. The integrated FL intensity of HSA–ICG NPs at different locations was translated into a mapping image (Figure 6D), indicating that the FL intensity collected from the tumor margin was much stronger than that of normal tissue, providing a wonderful description of the tumor. The precise identification of tumor position, size, and margin is liable to guide the laser probe to focus on the tumor tissue without injuring the normal tissue. The hematoxylin and eosin (H and E)-stained images of tumor sections collected from different treated groups of mice at 4 h post-injection via tail vein were obtained, and the HSA–ICG NP-treated group with continuous NIR laser irradiation (PDT/PTT) exhibited the most severe cancer necrosis among all groups (Figure 6E). All these results substantiated HSA–ICG NPs as the ideal candidate for synergetic PDT/PTT of cancers guided with dual-modal imaging and tumor margin detection (Sheng et al., 2014). In another study, Chen and coworkers designed an NIR dye (IR825)-loaded HSA complex, which showed highly intensive fluorescence intensity under 600-nm excitation and strong absorbance at 820 nm. The separated imaging and therapy wavelength channels rendered the HSA–IR825 complex the ideal candidate for effective PTT with imaging guidance (Chen et al., 2014). Tian et al. (2019) employed BSA to fabricate a tumor pH-responsive polyaniline (PANI)-based theranostic agent through intermolecular acid–base reactions between imine moieties of PANI and carboxyl groups of BSA. The synthesized BSA-PANI assemblies were demonstrated to be able to induce the red shift of absorbance from visible light region to near-infrared region in the acidic microenvironment of tumors with pH <7, while the PANI requires a more acidic environment with pH <4 to go through this process. The BSA-PANI assemblies exhibited the pH-responsive PA imaging property and enhanced PTT efficacy for tumors, rendering them the ideal candidate for PA imaging guided augmented PTT in the acidic tumor microenvironment.

### Sonodynamic Therapy-Based Cancer Theranostics

**FIGURE 7:**
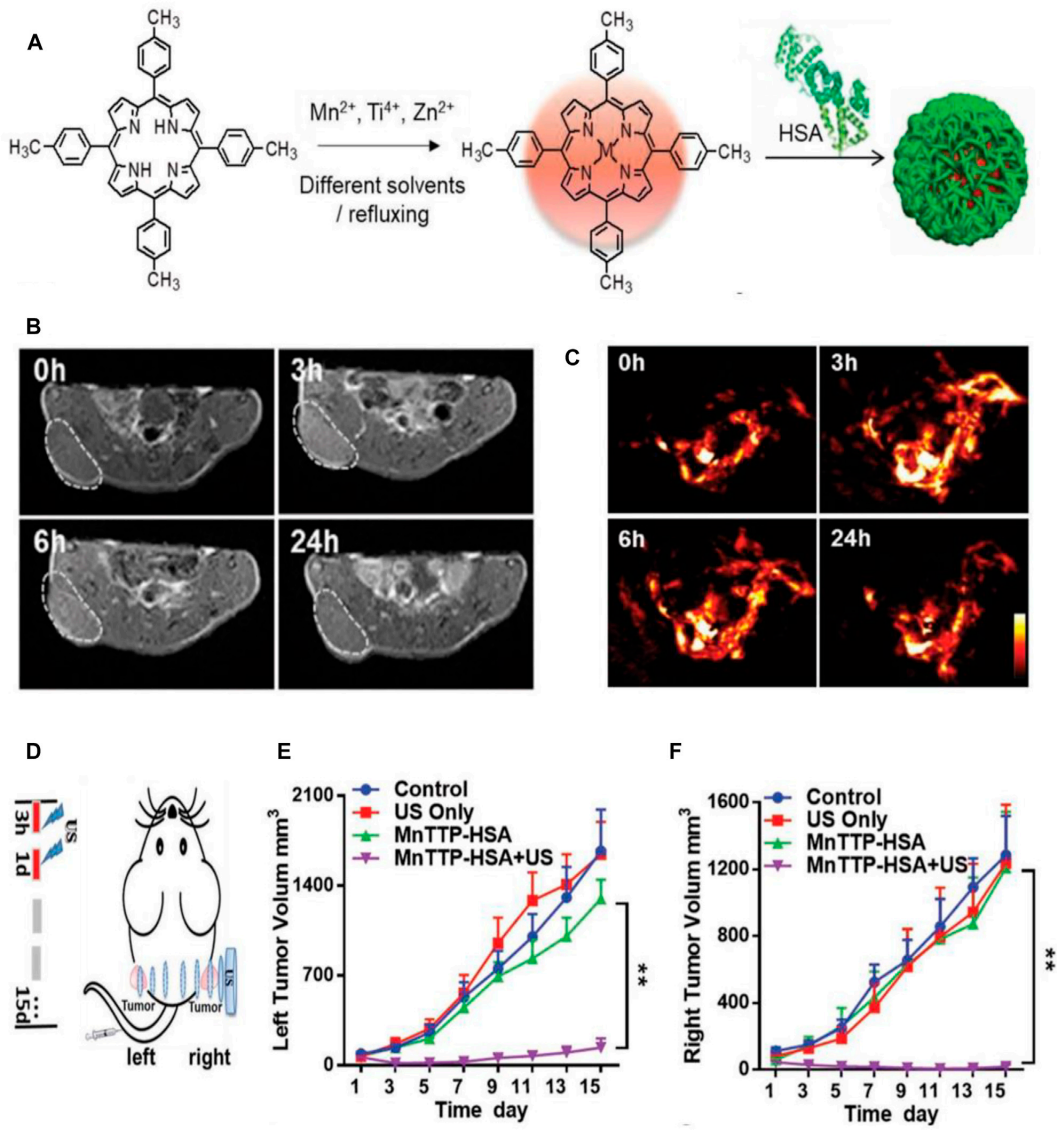
Sonodynamic therapy (SDT)-based cancer theranostics. **(A)** Schematic illustration of the synthesis procedure of three metal 4-methylphenylporphyrin (TTP) complexes (MnTTP, ZnTTP, and TiOTTP) and the corresponding nanocomplexes of MTTP–HSA. **(B)** The *in vivo* T_1_-weighted MR images of MCF-7 tumor-bearing nude mice after the i.v. injection of MnTTP–HSA. **(C)** The *in vivo* PA images of MCF-7 solid tumors after i.v. injection of MnTTP–HSA. **(D)** The *in vivo* SDT protocol of MCF-7 tumor-bearing mouse model with bilateral tumors. The US probe was placed on the right tumor, and the US irradiation was conducted twice at 3 and 24 h, respectively, from the right side to the left side (1.0 MHz, 2 W/ cm^2^, 50% duty cycle, 5 min). **(E, F)** The tumor growth curves of the left and right tumors, respectively, with various treatments (**p < 0.01). Reproduced with permission from Ma et al. (2019). Copyright 2018, WILEY-VCH Verlag GmbH and Co. KGaA, Weinheim.

In recent years, ultrasound-triggered sonodynamic therapy (SDT), as a rising star in nanomedicine, has attracted the attention of more and more researchers due to its noninvasiveness, higher tissue penetration depth, and fewer side effects (Liang et al., 2020). Similar to the PDT process, the sonosensitizers can be activated by low-intensity ultrasound (US) and generate reactive oxygen species (ROS) for cancer therapy during SDT (Pan et al., 2018). Compared with PDT that uses light as the energy source, SDT uses US as the energy source to activate the sonosensitizers, and SDT possesses higher tissue penetration depth, which can be applied to the noninvasive treatment of deep tumors (McEwan et al., 2016). However, SDT suffers from some inherent limitations, such as the poor tumor accumulation of small-molecule sonosensitizers and the lower quantum yield of inorganic sonosensitizers (Liang et al., 2020). Nowadays, porphyrin and its derivatives are the most extensively used sonosensitizers in SDT due to their various unique properties (Park J. M. et al., 2021). Nevertheless, the small-molecule porphyrins have low chemical and biological stability and are toxic to sensitive skin, which may lead to decreased therapeutic efficacy (Ma et al., 2019). It is worth noting that the metalloporphyrin complexes have been validated to have the ability to optimize these properties, but their low water solubility, rapid metabolism, and potential photosensitive toxicity hinder their wide application in cancer theranostics (Huang et al., 2017; Ma et al., 2019). Considering these situations, Ma and their coworkers synthesized three metal 4-methylphenylporphyrin (TTP) complexes (MnTTP, ZnTTP, and TiOTTP), and utilize HSA to encapsulate them to form the novel nanosonosensitizers named as MTTP–HSA (Figure 7A), which exhibited the spherical morphology with the average diameter of around 60 nm. MTTP-HSAs have been proven to generate ^1^O_2_ upon US irradiation, indicating their great potential as the nanosonosensitizers for SDT. The ^1^O_2_ produced by three types of MTTP–HSAs (MnTTP–HSA, ZnTTP–HSA, and TiOTTP–HSA) can be monitored even up to 11 cm away from the US probe, which is significant to the SDT of deep-seated tumors. MnTTP–HSA was proven to be easily excited under energy when compared with ZnTTP–HSA and TiOTTP–HSA, indicating the strongest ability of ROS generation of MnTTP–HSA. To assess the MR and PA imaging properties of MnTTP–HSA *in vivo*, MCF-7 tumor-bearing nude mice were i.v. administered with MnTTP–HSA, and the MR and PA images were obtained at pointed times (Figures 7B, C). The strongest T_1_-positive signal at the tumor region could be observed at 3 h post-injection, which was similar to when the highest PA signal appeared in PA imaging. The *in vivo* SDT efficiency of MnTTP–HSA against tumors was further evaluated by establishing the MCF-7 tumor-bearing mouse model with bilateral tumors. The US irradiation was conducted twice at 3 and 24 h, respectively, from the right side to the left side (Figure 7D). The tumor growth curves of left and right tumors revealed that the MnTTP–HAS-treated mice upon US irradiation exhibited a complete tumor growth inhibition (Figures 7E, F). The tumor growth on the left was almost completely suppressed mainly due to the high tissue penetration depth of US-activatable SDT, which penetrated the whole body of mice from the right tumors to the left ones. These results confirmed the MR/PA imaging properties and satisfactory SDT efficacy of MnTTP–HSA, realizing the monitoring of drug accumulation in real time for precise SDT of tumors (Ma et al., 2019). In another study, Wan and coauthors also reported a nanosonosensitizer based on HSA. They employed an assembly strategy to design the Mn^2+^-chelated HSA-chlorin e6 (Ce6) nanoassemblies (HCM NAs), which can be used as the targeting diagnostic and therapeutic agents for the combination of focused US-induced moderate thermal treatment (42°C) and SDT of gliomas in mice with fluorescence and MR imaging guidance (Wan et al., 2019). Xu et al. (2015) have developed tetra-α-(3-carboxyphenoxyl) zinc(II) phthalocyanine (ZnPcC_4_)-BSA conjugate to treat the HepG2 human hepatocarcinoma cells, which exhibited higher sonodynamic activity with an IC_50_ value of 7.5 μM when compared with ZnPcC_4_. The enhanced intracellular reactive oxygen species (ROS) level induced by ZnPcC_4_–BSA with exposure to ultrasound can lead to cellular apoptosis, suggesting the tremendous potential of ZnPcC_4_–BSA to serve as the sonosensitizers for SDT.

### Other Therapeutic Approach-Based Cancer Theranostics

Besides the mentioned serum albumin-based nanovehicle-assisted cancer theranostics, such as gas therapy, chemodynamic therapy (CDT), phototherapy (PTT/PDT), and sonodynamic therapy (SDT), serum albumin-based nanovehicles can be also used for other therapeutic approach-based cancer theranostics, involving chemotherapy, immunotherapy, radiotherapy, as well as cancer imaging. In an example of serum albumin loaded with chemotherapy drugs, HSA pre-modified with either chlorin e6 (Ce6) or cyclic Arg-Gly-Asp (cRGDyK) peptide was used to load PTX for tumor-targeted PDT and chemotherapy. The prominent tumor-targeted synergistic antitumor effect was successfully demonstrated both *in vitro* and *in vivo*. Remarkably, the almost tumor ablation can be observed in the group of mice treated with HSA-Ce6-PTX-RGD-1 upon 660-nm light irradiation. When labeled with Mn^2+^, these HSA-based PTX-loaded nanoparticles can be utilized as the contrast agents for T_1_-weighted MR and FL imaging of tumors. This work provided a brief approach to prepare the HSA-based cancer theranostic agents for tumor-targeted PDT and chemotherapy (Chen et al., 2015). To enhance the therapeutic effect of liver tumors, Qi et al. (2015) exploited recombinant human serum albumin (rHSA) covalently coupled with glycyrrhetinic acid (GA) as the drug delivery vehicle to deliver chemotherapeutic doxorubicin (DOX), which was physically incorporated into the nanoparticles (DOX/GA-rHSA NPs). DOX/GA-rHSA NPs exhibited a stronger antitumor activity and more endocytosis in liver tumor cells than the nontargeted NPs (DOX-loaded rHSA nanoparticles without GA conjugation). The *in vivo* biodistribution assay substantiated the enhanced tumor accumulation of DOX/GA–rHSA NPs at 1 h post-injection in hepatoma-bearing mice, demonstrating their liver-targeting capability and great potential in liver cancer therapy.

In the past few decades, PTT and PDT, used for cancer therapy, have received extensive attention due to their outstanding antitumor efficacy. However, tumor PTT and PDT still suffer from the high tumor recurrence rate and high risk of tumor metastasis (Rajendrakumar et al., 2018). Notably, phototherapy has been reported to induce antitumor immune responses by eliciting immunogenic cell death (ICD) of tumors (Chen et al., 2018). Therefore, with the aim of achieving a long-term therapeutic effect of phototherapy, Zhu and coauthors used BSA as the drug delivery vehicle to load photosensitizer Ce6 and aluminum adjuvant to form albumin-biomineralized nanoparticles (Al-BSA-Ce6 NPs), which have the ability to induce antitumor immune responses after photo tumor ablation and, thus, achieve a long-term therapeutic effect of phototherapy. The combined PTT and PDT triggered by Al-BSA-Ce6 NPs can destroy most primary tumors and release tumor-associated antigens, and finally induce the tumor antigen-specific cellular immune responses in the presence of aluminum adjuvant, playing the role of “*in situ* tumor vaccine.” When combined with CpG adjuvant, the generated antitumor immune responses were able to eradicate the remaining tumor cells within the primary tumor, inhibit tumor metastasis, and effectively reduce the risk of tumor recurrence. Unlike commercially available aluminum adjuvants that mainly induce humoral immune responses, the aluminum hydroxide core in Al-BSA-Ce6 NPs assisted the antigen to induce a strong cellular immune response, and this effect cannot be replaced by the ferric hydroxide core (Zhu et al., 2020). Owing to the utilization of albumin approved by the FDA and classic aluminum adjuvants, Al-BSA-Ce6 NPs has greater clinical transformation capability than other drug delivery vehicles, such as carbon nanotubes for photo-immunotherapy (Liang et al., 2014; Chen et al., 2016).

It has been reported that liposomes can quickly and massively enter tumor tissues by means of macrophage-induced vascular destruction under x-ray irradiation (Miller et al., 2017). However, the effect of x-ray irradiation on the *in vivo* transport of HSA is rarely studied. Therefore, Yi and coauthors studied the effect of X-ray irradiation on HSA transport, which was further applied to cancer theranostics. First, the expression of Caveolin-1 in cancer cells was confirmed to increase after exposure to x-rays both *in vitro* and *in vivo*, resulting in the enhanced cell uptake of HSA. Then HSA was labeled with radionuclide ^125^I to form ^125^I-HSA, which can be used as a wonderful contrast agent for SPECT/CT imaging of CT26 tumor-bearing mice. After the tumor was pre-irradiated with x-rays, the retention of ^125^I-HSA in tumor tissues became high, which is consistent with the conclusion that x-rays promote the endocytosis of HSA by tumor cells. Moreover, the severe tumor apoptosis can be observed by carrying out ^131^I-HSA-based radioisotope therapy (RIT) after external beam radiotherapy (EBRT). Additionally, in order to target the irradiated tumor, the GNQEQVSPLTLLKXC peptide (A15) was modified onto the ^131^I-HSA to target the thrombosis induced by x-ray exposure, leading to the improved therapeutic effect of a combination EBRT and RIT. This study provided an optimized strategy for SPECT/CT imaging-guided combination therapy based on HSA (Yi et al., 2020).

## Conclusion and perspectives

In this review, we summarize the recent advances on the applications of serum albumin-based nanovehicles in cancer diagnosis and therapy. We briefly introduce the drug-loading strategies of BSA and HSA, and discuss the different types of serum albumin-based nanovehicles including albumin nanoparticles, surface-functionalized albumin nanoparticles, and albumin nanocomplexes. Then we describe the application of these nanovehicles used as the nanoprobes in cancer diagnosis and emphasize the employment of serum albumin-based nanovehicles for cancer theranostics, involving gas therapy, chemodynamic therapy (CDT), phototherapy (PTT/PDT), sonodynamic therapy (SDT), and other therapies as well as cancer imaging. The mentioned drug delivery vehicles were shown to possess excellent biocompatibility, high stability, nontoxicity, non-immunogenicity, and brief preparation method, with the overall goal of realizing the enhanced antitumor effect with imaging guidance by the enhancement of drug accumulation in tumor tissues.

As the most abundant protein in plasma, serum albumin plays an essential role in regulating plasma colloidal osmotic pressure and transporting endogenous compounds, which possesses a stable structure and has good resistance to pH, temperature, and organic solvents. Compared with some polymer micelles or synthetic polymers, serum albumin is a natural polymer that does not need to be synthesized and has a stable source. The remarkable superiorities, such as high stability, good biocompatibility, nontoxicity, and non-immunogenicity, make serum albumin an ideal candidate for cancer theranostics. Rapidly growing tumors will ingest large amounts of albumin as a source of energy and amino acids through gp60 receptors and SPARC proteins; consequently, serum albumin-based nanovehicles can target tumor tissues through this way and the EPR effect. In addition, the various functional groups of albumins can be modified with targeting ligands to further achieve tumor targeting or enhanced tissue penetration. The hydrophobic, hydrophilic domains and the large number of functional groups available in the primary structure of serum albumin allow them an ideal candidate for drug delivery vehicle fabrication and delivering desired drugs.

Serum albumin is generally considered as safe by FDA. The albumin-bound formulation of paclitaxel, Abraxane, has been already approved by the FDA for the clinical treatment of metastatic breast cancer (Tomao et al., 2009). Of note, safety is the essential concern for clinical translation of serum albumin-based nanovehicles. Meanwhile, the synthesis of serum albumin-based nanovehicles are simple; most of serum albumin-based nanovehicles are synthesized by self-assembly. The simple synthesis and safety make serum albumin-based nanovehicles stand out from other nanoplatforms, such as graphene oxide and carbon nanotubes. More importantly, the functional interaction with the FcRn-mediated recycling pathway makes albumin have a long circulatory half-life, which can be employed to prolong the circulatory half-life of drugs that loaded onto the albumin (Sleep et al., 2013). Therefore, serum albumin may be the primary choice for drug delivery instead of other materials. Although albumin is an excellent carrier material for drug delivery vehicle preparation, their clinical application remains limited due to the limited source of HSA and the mild immune response of BSA. In recent years, recombinant human serum albumin (rHSA) expressed by yeast cells has similar biocompatibility and pharmacokinetic processes to HSA, and can be used as a substitute for HSA in the preparation of serum albumin-based nanovehicles (Zhu et al., 2018).

Exploitation of serum albumin for diagnostic and therapeutic drug delivery is a promising antitumor strategy, which can accurately locate and eliminate the solid tumors and reduce the risk of recurrence and metastasis. However, there are still obstacles in the application of serum albumin-based nanovehicles in cancer diagnosis and therapy. For example, the efficacy of PDT will decrease with the increase in oxygen consumption during PDT. Developing a strategy that can provide sufficient oxygen for the consumption of photosensitizers and continuously generate ROS has proven to be a hopeful approach to improve the efficacy of PDT (Tang et al., 2018). In addition, the insufficient intracellular content of H_2_O_2_ limits the therapeutic effect of CDT due to the unsustainable production of cytotoxic hydroxyl radical (•OH) via Fenton reaction. The main reason for this phenomenon is that the catalase expressed in various tumors can decompose H_2_O_2_ and reduce the production of cytotoxic hydroxyl radical (•OH), consequently leading to the compromised CDT. Therefore, developing an H_2_S gas-amplified ROS-based therapeutic nanovehicle for enhanced CDT is a promising antitumor approach, which is related to the inhibition effect of H_2_S molecules on catalase activities (Xie et al., 2020). Notably, SDT uses US as the energy source to activate the sonosensitizers and possesses higher tissue penetration depth when compared with PDT that uses light as the energy source, which is more suitable for the noninvasive treatment of deep tumors (Ma et al., 2019). For cancer treatment, monotherapy often has limited therapeutic effects on tumors, while synergistic therapy can achieve significantly enhanced therapeutic effects. Moreover, employing synergistic therapy can overcome the tumor tolerance to monotherapy. For imaging, multimodal imaging is able to overcome false positives that may be caused by single-mode imaging and improve the accuracy of imaging and detection. Furthermore, various imaging modes have their own unique advantages and scope of application, for example, X-ray imaging is good for skeletal structure imaging, while MRI is good for soft tissue imaging. Therefore, the utilization of multimodal imaging can realize complementary advantages and achieve better imaging effects.

Although a large number of serum albumin-based nanovehicles have been developed for cancer diagnosis and treatment, whether they can be produced on a large scale with the uniform and stable diameter distribution as well as high drug loading rate is an urgent problem to be confirmed. Different preparation conditions have significant effects on the diameter distribution and drug loading rate in the process of developing serum albumin-based nanovehicles. We hope that the clinical translation of serum albumin-based nanovehicles can be further accelerated with the continuous optimization of preparation technology and the control of manufacturing quality, which will enable nanovehicles, based on serum albumin, to play an indispensable role in tumor theranostics and generate economic value.
